# Nafamostat reduces systemic inflammation in TLR7-mediated virus-like illness

**DOI:** 10.1186/s12974-021-02357-y

**Published:** 2022-01-06

**Authors:** Abi G. Yates, Caroline M. Weglinski, Yuxin Ying, Isobel K. Dunstan, Tatyana Strekalova, Daniel C. Anthony

**Affiliations:** 1 Department of Pharmacology, The University of Oxford, Mansfield Road, Oxford, UK; 2 School of Biomedical Sciences, The University of Queensland, Brisbane, Australia; 3 Sechenov First Moscow State Medical University, Moscow, Russia; 4 Institute of General Pathology and Pathophysiology, Moscow, Russia; 5 University of Southern Denmark, Odense, Denmark

**Keywords:** Nafamostat, Viral infection, Inflammation, Sickness behaviour, COVID-19

## Abstract

**Background:**

The serine protease inhibitor nafamostat has been proposed as a treatment for COVID-19, by inhibiting TMPRSS2-mediated viral cell entry. Nafamostat has been shown to have other, immunomodulatory effects, which may be beneficial for treatment, however animal models of ssRNA virus infection are lacking. In this study, we examined the potential of the dual TLR7/8 agonist R848 to mimic the host response to an ssRNA virus infection and the associated behavioural response. In addition, we evaluated the anti-inflammatory effects of nafamostat in this model.

**Methods:**

CD-1 mice received an intraperitoneal injection of R848 (200 μg, prepared in DMSO, diluted 1:10 in saline) or diluted DMSO alone, and an intravenous injection of either nafamostat (100 μL, 3 mg/kg in 5% dextrose) or 5% dextrose alone. Sickness behaviour was determined by temperature, food intake, sucrose preference test, open field and forced swim test. Blood and fresh liver, lung and brain were collected 6 h post-challenge to measure markers of peripheral and central inflammation by blood analysis, immunohistochemistry and qPCR.

**Results:**

R848 induced a robust inflammatory response, as evidenced by increased expression of TNF, IFN-γ, CXCL1 and CXCL10 in the liver, lung and brain, as well as a sickness behaviour phenotype. Exogenous administration of nafamostat suppressed the hepatic inflammatory response, significantly reducing TNF and IFN-γ expression, but had no effect on lung or brain cytokine production. R848 administration depleted circulating leukocytes, which was restored by nafamostat treatment.

**Conclusions:**

Our data indicate that R848 administration provides a useful model of ssRNA virus infection, which induces inflammation in the periphery and CNS, and virus infection-like illness. In turn, we show that nafamostat has a systemic anti-inflammatory effect in the presence of the TLR7/8 agonist. Therefore, the results indicate that nafamostat has anti-inflammatory actions, beyond its ability to inhibit TMPRSS2, that might potentiate its anti-viral actions in pathologies such as COVID-19.

## Introduction

The serine protease inhibitor nafamostat is in clinical trials as a potential treatment for COVID-19, owing to its ability to inhibit TMPRSS2-mediated viral entry of SARS-CoV2 into lung epithelial cells [1, 2]. Indeed, nafamostat was identified as a potential therapy using a Dual Split Protein reporter fusion assay, to screen a library consisting of 1017 FDA-approved drugs that were able to prevent S protein-initiated membrane fusion of MERS-CoV, a similar coronavirus, from infecting cells in this way [3]. This screening result, together with experimental data from MERS-CoV infection of cultured airway epithelial cell-derived Calu-3 cells, highlighted that nafamostat, over all other screened compounds, could be effective at inhibiting viral entry. In addition to its anti-viral properties, nafamostat is an inactivator of coagulation fibrinolysis, and platelet aggregation with potent inhibitory activity against thrombin, coagulation factors in active form (XIIa, Xa), kallikrein, plasmin, and complement factors (Clr, Cls). For this reason, nafamostat is used to treat disseminated intravascular coagulation (DIC) routinely in Japan [4]. However, it has also been shown to improve outcomes in models of stroke [5] and of spinal cord injury [6], and it is also used to treat pancreatitis [7], suggesting it has additional, immunomodulatory effects, which are beneficial to the host. However, to date, its impact on virally induced inflammation is unclear.

Current models of systemic inflammation often employ bacteria-derived polysaccharides such as LPS from *E. coli* or *S. enterica.* These activate the pattern recognition receptor (PRR) toll-like receptor 4 (TLR4), which, via myeloid differentiation primary response (MyD) 88, causes NF-κB translocation to the nucleus and upregulation of central and peripheral cytokine expression [8]. The host response to TLR agonists has evolved to recognise and protect the individual from the full spectrum of infectious agents. A common feature of TLR activation is the acute-phase response (APR) and the generation of liver-derived acute-phase proteins (APP), which mobilise leukocytes, such as neutrophils from the spleen and bone marrow into the circulation, and then to the site of infection to eliminate the pathogen [9]. The expression profile of the APPs is dependent on the nature of the pathogen and the TLR signalling pathways that activated. Typically, the APR is associated with the production of pentraxins, such as C-reactive protein (CRP), serum amyloid-A (SAA), and the expression of cytokines such as IL-1β, TNF and IL-6, and chemokines such as CXCL1, CCL2 and CXCL10 [10]. However, the production of peripheral cytokines is also associated with de novo cytokine expression in the brain. As a consequence, behavioural responses, termed sickness behaviours, are induced owing to the expression of central cytokines [11]. These behaviours are characterised by reduced activity, anorexia, and decreased motivational and goal-directed activity [12]. These inflammatory and behavioural responses are evident following both bacterial and viral infection [13], however, the responses are coordinated via different signalling pathways [14].

RNA viruses are detected by PRRs recognising either single-stranded RNA (ssRNA), such as SARS-CoV-2, or double-stranded RNA (dsRNA), such as reoviruses [15]. ssRNA and dsRNA are detected by TLR7/8 or TLR3, respectively, and trigger distinct downstream responses. The TLR3 pathway is MyD88-independent and induces a type-I interferon response, driven largely by the transcription factor IRF3 and designed to induce the proliferation of antigen-specific CD8+ T-cells. The inflammatory response to TLR7/8 stimulation also induces a type-1 interferon response, but it is initiated by the transcription factor IRF7, which is expressed at much lower levels than the more ubiquitous IRF3 [16]. Activation of TLR7 triggers a MyD88-dependent cascade, involving interleukin-1 receptor-associated kinase 1 (IRAK-1), IRAK-4, and TNF receptor-associated factor 6 (TRAF6), which leads to NFkB and IRF7 activation [14]. Phosphorylated IRF-7 then upregulates the production of the type-I interferons (IFNs), such as IFN-α and IFN-β. Although stimulation of both TLR3 and TLR7/8 induces inflammation, the differences in the underlying mechanisms are large enough to justify exploration of the consequence of a TLR7/8-mediated event, independent of TLR3 signalling in vivo.

In vivo models of viral infection use molecules specifically designed to target PRRs. This approach is safer than using live or attenuated viruses, avoiding the associated health risks, and allows greater control of the inflammatory response with dosage manipulation. The most common model uses polyinosinic:polycytidylic acid (polyI:C), a synthetic dsRNA, which thus stimulates TLR3 [13]. However, this fails to replicate the stimulation which occurs from ssRNA viruses such as SARS-CoV2. ssRNAs, such as ssRNA40, have been used previously to induce TLR7 signalling for the study of downstream inflammation [17–19], however, not in the context of mimicking viral infection. In addition, owing to the labile nature of RNA, its half-life in vivo is too short to be useful in understanding systemic effects [20, 21]. Therefore, a more robust TLR7-mediated inflammatory model is needed. R848 (resiquimod), is known to strongly stimulate TLR7, but not TLR8, in mice [22]. Whilst R848 has been used to replicate virus-induced TLR7 signalling previously [23, 24], studies evaluating its potential to simulate acute sickness behaviour are limited [22, 25]. Therefore, the effects of R848, in terms of both the systemic and central inflammatory responses, and the behavioural effects of TLR7/7 in mice stimulation require further characterisation.

The principal aims of this paper were to investigate the effects of R848 on acute behaviour and the systemic and central inflammatory responses, as well as to evaluate the potential for nafamostat to counteract the actions of TLR7 activation in mice.

## Methods

### Animals

Male CD-1 mice, 9 weeks of age (39–40 g) were purchased from Charles River (Oxford, UK) and allowed to acclimatise for 5 days before experimentation. Animals were housed in a pathogen-free facility, under standard diurnal lighting conditions (lights on 6 am–6 pm) with ad libitum access to water and standard chow (Global diet 2916C, Envigo), consisting of proteins, oils and fats, fibres and nutritional additives (Vitamins A and D_3_, Fe, Mn, Zn, Cu, I). Two cohorts were used for this study; one cohort was used for behavioural analysis and the other was used for leukocyte and qPCR analysis. Animals for behavioural analysis were given water with 1% sucrose during acclimatisation to train for sucrose preference test (SPT). Animals were housed *n* = 4 per cage, but separated and individually housed on the day of the experiment. All experiments were approved by the University of Oxford local committees (LERP, ACER) in accordance with the UK Animals (Scientific Procedures) Act 1989 and were performed in compliance with the ARRIVE guidelines.

### Challenge

Stock R848 (resiquimod, Enzo Life Sciences, UK) was prepared by diluting in dimethyl sulfoxide (DMSO) (Sigma-Aldrich, UK) to a concentration of 10 mg/mL. Stock R848 was then diluted 1:10 in sterile saline to 1 mg/mL and 200 μL of the working solution was injected intraperitoneally (200 μg of R848 per mouse). Control animals received a 200 μL intraperitoneal injection of the vehicle DMSO diluted 1:10 in sterile saline. Naïve animals that received no injections were used to establish baseline. All animals were culled 6 h post-challenge.

### Nafamostat treatment

100 μL of nafamostat (3 mg/kg) (Sigma-Aldrich, UK) in 5% dextrose was injected intravenously at the time of challenge. Dose is consistent with previous publications reporting beneficial effects [26–28]. Control animals received an intravenous injection of 5% dextrose alone.

### Behaviour

**Fig. 1:**
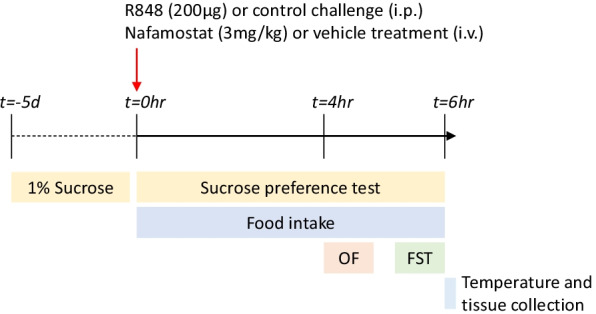
Schematic of behavioural tests performed. In summary, animals were exposed to 1% sucrose solution during the 5 days of acclimatisation. On the day of the experiment, male, CD-1 mice received an intraperitoneal injection of R848 (200 μg, dissolved in DMSO and sterile saline) or control solution (DMSO diluted in sterile saline), together with an intravenous injection of nafamostat (3 mg/kg) or vehicle (sterile saline). For the duration of the experiment, animals underwent the sucrose preference test (SPT), and food intake was examined. 4-h post-challenge, animals completed the open field (OF) paradigm. 6 h post-challenge, animals completed the forced swim test (FST), after which their temperature was determined and animals were culled. d = days, hr = hours

To determine the effect of R848 and nafamostat on sickness behaviour, food intake during the 6-h experiment was recorded. Immediately after the challenge, mice were given a mass of food, which was weighed before (t0) and after (at 6 h) the experiment. The difference in weight, indicating that consumed by the animals, was calculated. In addition, SPT, open field (OF) and forced swim test (FST) were performed. All behavioural tests were completed during the dark cycle, in line with previous publications [29, 30]. For SPT, animals were given access to 2 bottles, one containing normal water and one containing 1% sucrose, for the 6-h duration of the experiment. To prevent location bias, bottles were switched 3-h post-challenge [12]. Total sucrose water intake (mL) and sucrose preference (volume [sucrose water]/volume[sucrose + normal water] × 100) were determined. OF analysis was performed 4-h post-challenge. Animals were placed in the corner of the apparatus and allowed to explore for 5 min. The number of grid crossings and rears, and time spent in the centre were recorded. For the FST [31], the animals were placed into water maintained at 29–31 °C to avoid bias caused by invigorating effects of cold water and allowed to swim for 5 min. For analysis, the first 2 min of swimming was disregarded; the latency to float and total float time were measured. OF and FST analyses were completed in a blinded fashion. Summary of behaviour tests completed is visualised in Fig. 1.

### Temperature and tissue collection

Mice were anaesthetised with isoflurane and surface temperature was determined with FLIR B360 thermal imaging camera (FLIR Systems, UK). Blood was then collected by cardiac puncture, transferred into an EDTA-coated tube, and immediately measured for leukocyte analysis. Animals were then intracardially perfused with cold, heparinised saline, and fresh liver, lung and brain were collected and snap-frozen.

### Blood analysis

Approximately 200 μL of blood was collected for leukocyte analysis. Within 20 min of collection, blood was measured in triplicate on the ABX Pentra 60 (Horiba, UK). Total leukocyte concentration, and % lymphocytes, monocytes, neutrophils, basophils and eosinophils were recorded. Data were analysed and presented as 10e^3^ cells per μL of blood (K/μL).

### RNA extraction and cDNA conversion

RNA was extracted from fresh liver, lung and prefrontal cortex of the brain, using the Qiagen RNeasy Mini kit, according to manufacturer’s instructions. RNA concentration was measured using a NanoDrop (Thermo Scientific, UK), and 1000 ng of RNA was converted to cDNA with the High Capacity cDNA conversion kit (Applied Biosystems), following the manufacturer’s instructions.

### Immunohistochemistry and quantification

Immunohistochemistry was performed with the chromogenic reporter 3,3′-diaminobenzidine (DAB). Fresh tissue was cut at 12 μm with the Leica cryostat and post-fixed in 2% PFA for 1 h. After a brief wash in PBS, endogenous peroxidase activity was quenched with 1% H_2_O_2_ and blocking for endogenous avidin and biotin (1:20, Vector Labs) and non-specific binding (10% goat serum) was performed. Sections were then incubated in primary antibody (rabbit α-Iba-1, AbCam, 1:2000; rabbit α-MBS [neutrophils], 1:10,000, made in house; rat α-B220, 1:900, BD Pharmingen) overnight at 4 °C. The following day, sections were incubated in biotinylated secondary antibody (goat α-rabbit or goat α-rat, 1:200, Vector Labs) for 2 h at RT, followed by ABC (1:100, ThermoFisher) for 1 h at RT. Sections were washed and incubated in DAB until a satisfactory level of staining was achieved, and 1% haematoxylin was used as a counterstain. Stained sections were dehydrated through graded alcohols (80%, 95%, 2 × 100%), cleared with xylene and mounted with DPX.

For quantification, stained cells were counted directly using a light microscope (Leitz Dialux 20) with an eyepiece grid at 40× magnification. Counts were performed across three sections, in three representative fields per section. In the brain, cell counts were performed in the prefrontal cortex, specifically, due to its association with inflammation and sickness behaviour [12, 32, 33]. All cell counting was performed blinded, and results are presented as cells/mm^2^.

### qPCR

Real-time qPCR was performed with samples in duplicate (25 ng/well) using SYBR green qPCR master mix (PrimerDesign) with the Roche LightCycler 480. Primers for TNF (F: AGCCAGGAGGGAGAACAGA, R: CAGTGAGTGAAAGGGACAGAAC), IFN-γ (F: ACAGCAAGGCGAAAAAGGATG, R: TGGTGGACCACTCGGATGA), CXCL1 (F: GCTGGGATTCACCTCAAGAAC, R: TGTGGCTATGACTTCGGTTTG), CXCL10 (F: CATCCCGAGCCAACCTTCC, R: CACTCAGACCCAGCAGGAT), SAA-2 (F: TGGCTGGAAAGATGGAGACAA, R: AAAGCTCTCTCTTGCATCACTG) and CRP (F: GGGTGGTGCTGAAGTACGAT; R: CCAAAGACTGCTTTGCATCA) were purchased from Sigma. Relative expression was determined by the 2^−ΔΔCT^ method, normalised to GAPDH as the housekeeping gene (PrimerDesign).

### Statistical analysis

All statistical analyses were completed with GraphPad Prism 7 software. Statistical outliers as determined by the software, were excluded from analysis. Two-way analysis of variance (ANOVA) was employed, with Sidak’s post hoc test as appropriate. Results were considered significant at *p* < 0.05 with 95% confidence intervals. All quantitative data are expressed as mean ± standard error of the mean (SEM).

## Results

### R848 induces sickness behaviour in mice, which is not attenuated by nafamostat

**Fig. 2:**
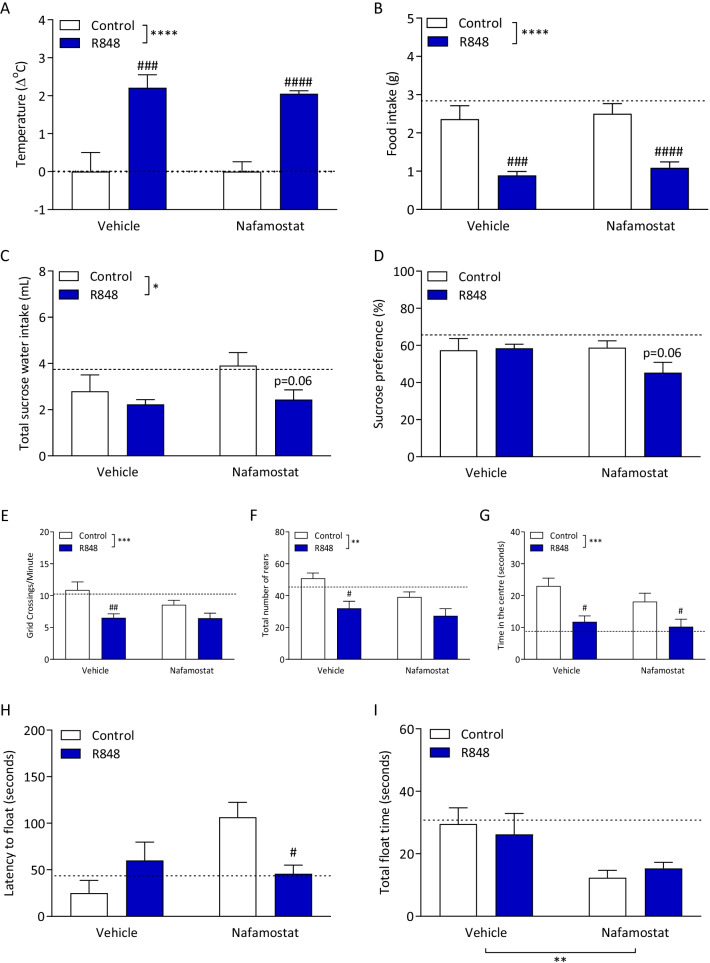
R848 induces a sickness behaviour phenotype, which is not ameliorated by nafamostat. Male, CD-1 mice received an intraperitoneal injection of R848 (200 μg, dissolved in DMSO and sterile saline) or control solution (DMSO diluted in sterile saline), together with an intravenous injection of nafamostat (3 mg/kg) or vehicle (sterile saline), and sickness behaviour was assessed 4–6 h later. Animal temperature (**A**) and food intake (**B**) were determined to assess any fever and anorexia, respectively. Anhedonia was measured with the SPT; total sucrose water intake (**C**) and preference for the sucrose water (**D**) were evaluated. OF was used to assess exploratory behaviour and grid crossings/minute (**E**), number of rears (**F**) and time spent in the centre (**G**) were calculated. Latency to immobility (**H**) and total immobility (**I**) in the FST were measured. Naïve animals were included to establish baseline (dotted line). Data presented as mean ± SEM, *n* = 4–10/group, and analysed by two-way ANOVA, *p < 0.05, **p < 0.01, ***p < 0.001, ****p < 0.0001 main effect, ^#^*p* < 0.05, ^##^*p* < 0.01, ^###^*p* < 0.001, ^####^*p* < 0.0001 control vs. R848 in Sidak’s post hoc test

In this study, we investigated whether R848 administration would mimic the sickness behaviours associated with viral infection [34, 35]. Following the R848 challenge, animal temperature was significantly increased, indicative of a fever (Fig. 2A; two-way ANOVA, challenge *p* < 0.0001). There was no main effect of the nafamostat (*p* = 0.788) and no significant interaction (*p* = 0.788). R848 induced a decrease in food intake over the 6-h experiment (Fig. 2B; two-way ANOVA, challenge *p* < 0.0001). Sidak’s post hoc testing confirmed significant differences between the control and the R848-treated animals (vehicle, *p* < 0.001; nafamostat, *p* < 0.0001), independent of nafamostat treatment (main effect, *p* = 0.439; interaction, *p* = 0.889).

Anhedonia, the compromised ability to experience pleasure, was determined by the SPT. There was a main effect of the R848 challenge on total sucrose intake (Fig. 2C; two-way ANOVA, challenge *p* < 0.05), but no effect of nafamostat (*p* = 0.204) and no interaction (*p* = 0.382). When intake of sucrose water was assessed as a preference of total fluid intake, there was no main effect of the challenge or the treatment, and no interaction (Fig. 2D; two-way ANOVA, interaction *p* = 0.126, challenge *p* = 0.192, treatment *p* = 0.214).

The OF paradigm was used to measure exploratory behaviour, which is known to be affected by sickness behaviour—an increase in activity can be interpreted as a measure of pleasurable activity in rodents and a decrease is regarded as a sign of a depressive-like behaviour [35, 36]. Following R848 administration, the number of grid crossings/minute (Fig. 2E; two-way ANOVA, challenge *p* < 0.001) and the total number of rears (Fig. 2F; two-way ANOVA, challenge *p* < 0.01) were significantly decreased. These behavioural changes were not altered by nafamostat and there were no significant interactions. Animals challenged with R848 also spent less time in the centre of the apparatus (Fig. 2G; two-way ANOVA, challenge *p* < 0.001); there was no significant effect of the nafamostat (*p* = 0.210) and no significant interaction (*p* = 0.516).

In the FST, there was no main effect of R848 challenge or nafamostat treatment on the latency to float (Fig. 2H; two-way ANOVA, challenge *p* = 0.476, treatment *p* = 0.067). However, there was a significant interaction (*p* < 0.05); R848 induced a non-significant increase in latency to float in vehicle-treated animals. In nafamostat-treated animals, R848 induced a significant decrease (Sidak’s post hoc test, *p* < 0.05). R848 had no effect on the total float time (Fig. 2I; two-way ANOVA, challenge *p* = 0.979), but nafamostat had a main treatment effect (*p* < 0.01). There was no significant interaction (*p* = 0.532).

### R848 depletes circulating leukocytes, which is partially restored by nafamostat

**Fig. 3:**
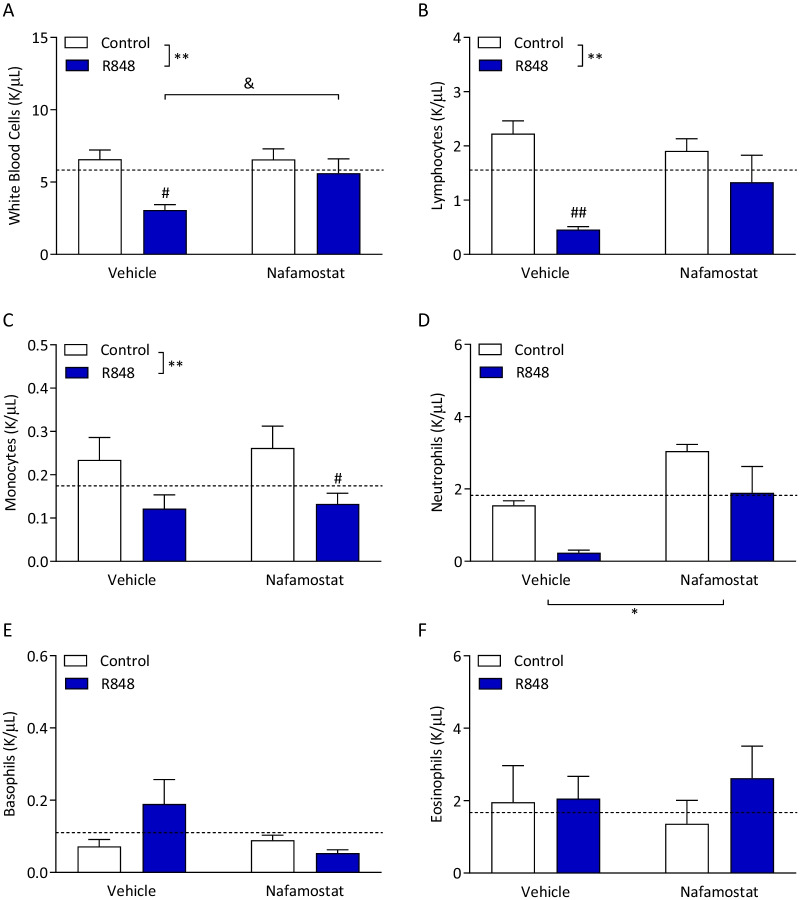
R848 causes a depletion in circulating leukocytes, which is partially restored by nafamostat. Male, CD-1 mice received an intraperitoneal injection of R848 (200 μg, dissolved in DMSO and sterile saline) or control solution (DMSO diluted in sterile saline), together with an intravenous injection of nafamostat (3 mg/kg) or vehicle (sterile saline). Blood was collected 6-h post-challenge and total concentration of total white blood cells (**A**), as well as the concentrations of lymphocytes (**B**), monocytes (**C**), neutrophils (**D**), basophils (**E**) and eosinophils (**F**), specifically, were measured. Naïve animals were included to establish baseline (dotted line). Data presented as mean ± SEM, *n* = 4–10/group, and analysed by two-way ANOVA, **p < 0.01 main effect, ^#^*p* < 0.05, ^##^*p* < 0.01 control vs. R848 with Sidak’s post-hoc test, ^&^*p* < 0.05 vehicle vs. nafamostat with Sidak’s post-hoc test

Analysis of the peripheral leukocyte populations revealed that R848 induced leukopenia (Fig. 3A; two-way ANOVA, interaction *p* = 0.114, challenge *p* < 0.01, treatment *p* = 0.115). In vehicle-treated animals, total blood leukocytes were significantly decreased with R848 challenge compared to control (Sidak’s post hoc test, *p* < 0.05). This was normalised by nafamostat in the R848-challenged animals (Sidak’s post hoc test, p < 0.05). Changes in specific subtypes of leukocytes were then investigated, to explore those responsible for the total leukocyte count result. R848 administration induced a significant decrease in blood lymphocytes (Fig. 3B; two-way ANOVA, interaction *p* = 0.083, challenge *p* < 0.01, treatment *p* = 0.405). In post-hoc testing, challenge with R848 induced lymphocyte depletion in vehicle-treated animals (*p* < 0.01). There was no corresponding depletion in the nafamostat-treated animals (*p* = 0.339), although there was also no difference between the R848 groups (*p* = 0.102). Monocytes were significantly decreased in R848 animals, which was not ameliorated by nafamostat (Fig. 3C; two-way ANOVA, interaction *p* = 0.839, challenge *p* < 0.01, treatment *p* = 0.641). There was no main effect of the challenge on blood neutrophils (Fig. 3D; two-way ANOVA, challenge *p* = 0.106), however there was a nafamostat main effect (*p* < 0.05). No post-hoc tests were significant, and there was no significant interaction (*p* = 0.916). Blood basophils (Fig. 3E; two-way ANOVA, interaction *p* = 0.103, challenge *p* = 0.372, treatment *p* = 0.197) and eosinophils (Fig. 3F; two-way ANOVA, interaction *p* = 0.556, challenge *p* = 0.493, treatment *p* = 0.989) were unaffected by both the R848 challenge and nafamostat treatment.

**Fig. 4:**
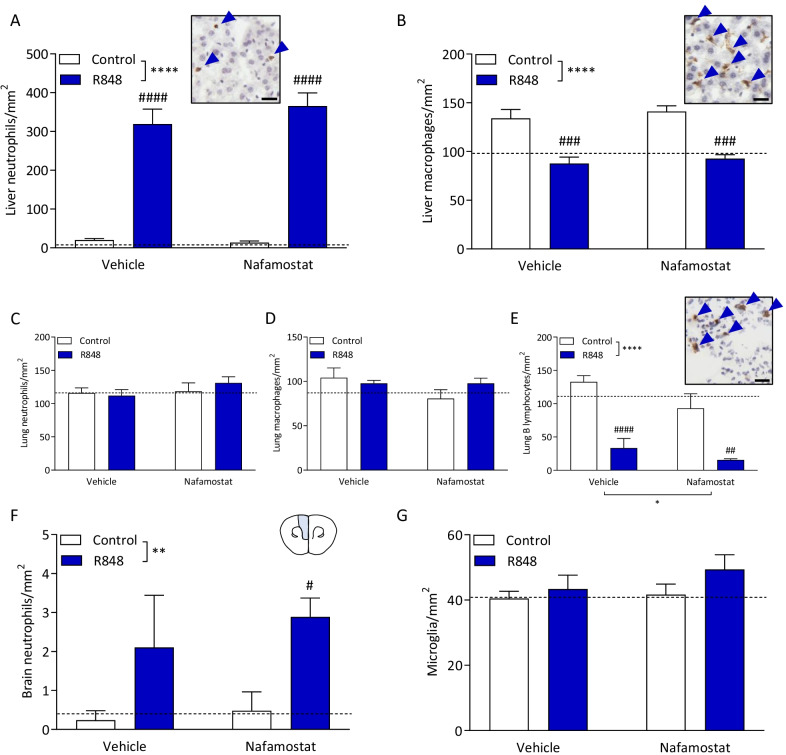
R848 induces neutrophil infiltration of peripheral and central tissues, but depletion of monocytes and B lymphocytes. Male, CD-1 mice received an intraperitoneal injection of R848 (200 μg, dissolved in DMSO and sterile saline) or control solution (DMSO diluted in sterile saline), together with an intravenous injection of nafamostat (3 mg/kg) or vehicle (sterile saline). Fresh liver was collected 6-h post-challenge and a subset of each group was cut at 12 μm thickness (*n* = 5/group). Immunostaining for neutrophils (MBS+), macrophages (Iba-1+) and B lymphocytes (B220+) was performed to determine leukocyte density. Liver neutrophils (**A**) and macrophages (**B**), lung neutrophils (**C**), macrophages (**D**) and B lymphocytes (**E**), and brain neutrophils (**F**) and microglia (**G**) were quantified, blinded, and presented as cells per mm^2^. Naïve animals were included to establish baseline (dotted line). Data presented as mean ± SEM and analysed by two-way ANOVA, ****p < 0.0001, **p < 0.01 main effect, ^#^*p* < 0.05, ^##^*p* < 0.01, ^###^*p* < 0.001, ^####^*p* < 0.0001 control vs. R848 with Sidak’s post hoc test

To determine whether the decrease in circulating leukocytes was due to sequestering into organs, immunostaining for neutrophils, macrophages and B lymphocytes in the liver, lung and brain was performed. Neutrophil infiltration of the liver is a common marker of systemic inflammation associated with infection [37]. Consistent with this, we show here that R848 challenge induced a significant increase in neutrophil density of the liver (Fig. 4A; two-way ANOVA, interaction *p* = 0.320, challenge *p* < 0.0001, treatment *p* = 0.454). By contrast, liver Kupffer cells were significantly decreased with the R848 challenge (Fig. 4B; two-way ANOVA, interaction *p* = 0.881, challenge *p* < 0.0001, treatment *p* = 0.374). For both cell types, there was no effect of nafamostat. A scattering of B220+ B lymphocytes were observed in the liver, which was not quantifiable due to the low number (data not shown). In the lung, both R848 and nafamostat had no effect on neutrophil (Fig. 4C; two-way ANOVA, interaction *p* = 0.454, challenge *p* = 0.697, treatment *p* = 0.334) and macrophage density (Fig. 4D; two-way ANOVA, interaction *p* = 0.131, challenge *p* = 0.472, treatment *p* = 0.131). However, the challenge did induce a significant decrease in B lymphocyte density (Fig. 4E; two-way ANOVA, interaction *p* = 0.408, challenge *p* < 0.0001). Moreover, there was a main effect of nafamostat treatment (treatment *p* < 0.05); fewer cells were observed in the nafamostat-treated animals compared with vehicle controls. In the prefrontal cortex of the brain (Fig. 4F, insert), R848 induced significant neutrophil recruitment (Fig. 4F; two-way ANOVA, interaction *p* = 0.692, challenge *p* < 0.01, treatment *p* = 0.458), but treatment with nafamostat had no effect. Brain microglial density in the prefrontal cortex was unaffected by R848 and nafamostat (Fig. 4G; two-way ANOVA, interaction *p* = 0.519, challenge *p* = 0.166, treatment *p* = 0.338). No B220+ B lymphocytes were detected in the brain (data not shown).

### R848 induces systemic inflammation that is attenuated by nafamostat

**Fig. 5:**
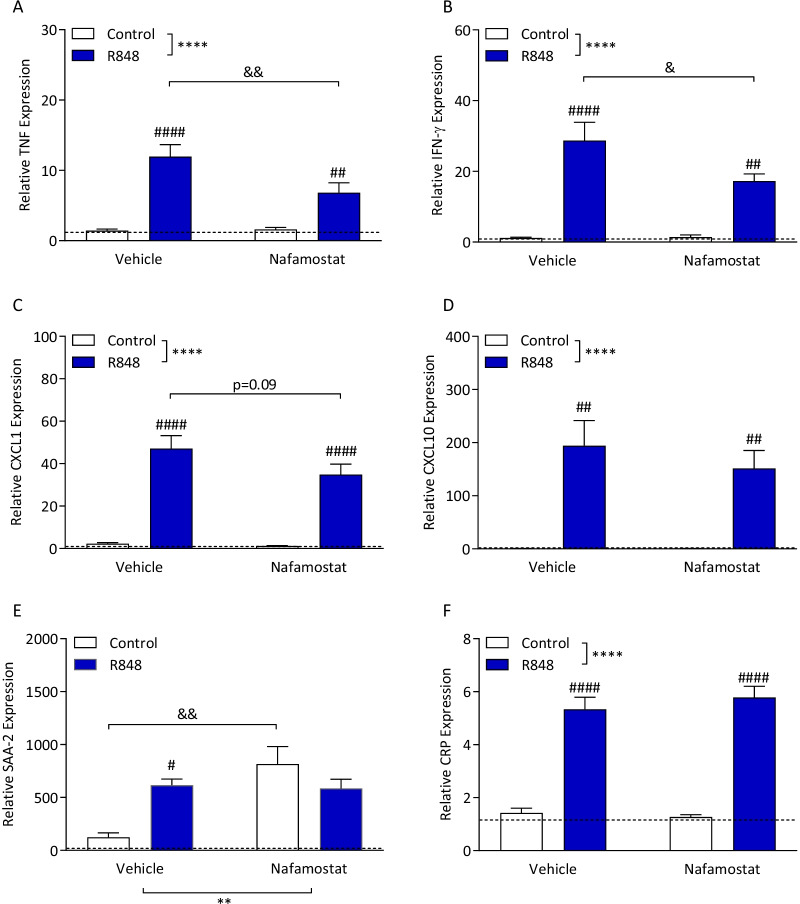
R848 induces pro-inflammatory gene expression in the liver, which is ameliorated with nafamostat treatment. Male, CD-1 mice received an intraperitoneal injection of R848 (200 μg, dissolved in DMSO and sterile saline) or control solution (DMSO diluted in sterile saline), together with an intravenous injection of nafamostat (3 mg/kg) or vehicle (sterile saline). Fresh liver was collected 6-h post-challenge and relative expression of TNF (**A**), IFN-γ (**B**), CXCL1 (**C**), CXCL10 (**D**), SAA-2 (**E**) and CRP (**F**) were determined by qPCR. Naïve animals were included to establish baseline (dotted line). Data presented as mean ± SEM, *n* = 4–10/group, and analysed by two-way ANOVA, ****p < 0.0001 main effect, ^##^*p* < 0.01, ^####^*p* < 0.0001 control vs. R848 with Sidak’s post hoc test, ^&^*p* < 0.05, ^&&^*p* < 0.01 vehicle vs. nafamostat with Sidak’s post hoc test

Previous work from our group has shown that the changes in the levels of mRNA for APPs correspond to changes in the protein levels of the APP [10, 38]; we measured mRNA levels to explore the impact of R848 and nafamostat on the inflammatory markers. In the liver, R848 induced a significant increase in the expression of TNF (Fig. 5A; two-way ANOVA, challenge *p* < 0.0001, treatment *p* = 0.075). An interaction between the R848 challenge and nafamostat treatment approached significance (*p* = 0.057); Sidak’s post-hoc test of challenged animals revealed a significant decrease in TNF expression with nafamostat treatment (p < 0.01). Similarly, hepatic IFN-γ expression was increased with R848 challenge (Fig. 5B; two-way ANOVA, interaction *p* = 0.098, challenge *p* < 0.0001, treatment *p* = 0.114), which was ameliorated by nafamostat (Sidak’s post hoc test, *p* < 0.05). R848 induced an increase in CXCL1 expression in the liver (Fig. 5C; two-way ANOVA, challenge *p* < 0.0001). However, there was no main effect of nafamostat (*p* = 0.166), and no interaction (*p* = 0.242). A trending decrease in the level of CXCL1 expression in R848 animals treated with nafamostat, compared with vehicle, was observed, although this was not statistically significant (Sidak’s post hoc test, *p* = 0.09). R848 had a main effect on hepatic CXCL10 expression (Fig. 5D; two-way ANOVA, challenge *p* < 0.0001), however there was no main effect of the nafamostat (*p* = 0.545) and no interaction (*p* = 0.543). Expression of APP SAA-2 was significantly perturbed in this study (Fig. 5E). Two-way ANOVA revealed a significant interaction (*p* < 0.01) with a main effect of nafamostat (*p* < 0.01), but not the challenge (*p* = 0.279). CRP was significantly increased in response to the R848 challenge (Fig. 5F; two-way ANOVA, challenge p < 0.0001), however, there was no main effect of treatment with nafamostat (*p* = 0.710) and no interaction (*p* = 0.437).

**Fig. 6:**
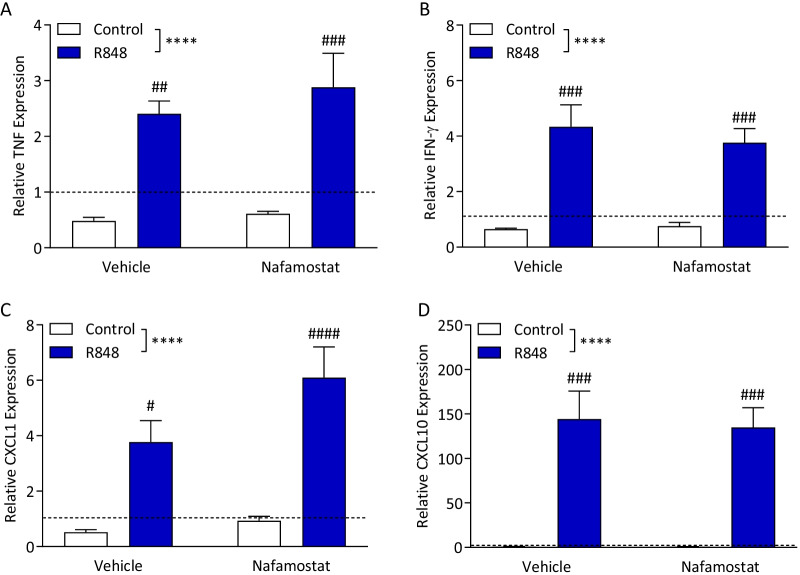
R848 induces pro-inflammatory gene expression in the lung, which is unaffected by treatment with nafamostat. Male, CD-1 mice received an intraperitoneal injection of R848 (200 μg, dissolved in DMSO and sterile saline) or control solution (DMSO diluted in sterile saline), together with an intravenous injection of nafamostat (3 mg/kg) or vehicle (sterile saline). Fresh lung was collected 6-h post-challenge and relative expression of TNF (**A**), IFN-γ (**B**), CXCL1 (**C**) and CXCL10 (**D**) was determined by qPCR. Naïve animals were included to establish baseline (dotted line). Data presented as mean ± SEM, *n* = 4–10/group, and analysed by two-way ANOVA, ****p < 0.0001 main effect, ^#^*p* < 0.05, ^##^*p* < 0.01, ^###^*p* < 0.001 control vs. R848 with Sidak’s post hoc test

In the lung, TNF (Fig. 6A; two-way ANOVA, interaction *p* = 0.666, challenge p < 0.0001, treatment *p* = 0.456), IFN-γ (Fig. 6B; two-way ANOVA, interaction *p* = 0.558, challenge *p* < 0.0001, treatment *p* = 0.684), CXCL1 (Fig. 6C; two-way ANOVA, interaction *p* = 0.255, challenge *p* < 0.0001, treatment *p* = 0.106) and CXCL10 (Fig. 6D; two-way ANOVA, interaction *p* = 0.841, challenge *p* < 0.0001, treatment *p* = 0.836) were all significantly upregulated compared to both control and naïve animals. Nafamostat had no effect on the expression of any genes analysed.

### R848 induces central inflammation which is unaffected by nafamostat treatment

**Fig. 7:**
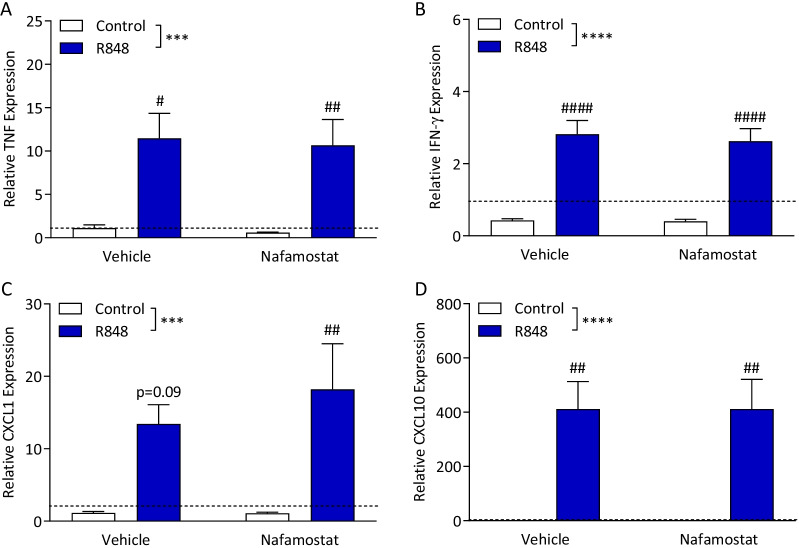
R848 causes pro-inflammatory gene expression in the brain which is not attenuated by nafamostat. Male, CD-1 mice received an intraperitoneal injection of R848 (200 μg, dissolved in DMSO and sterile saline) or control solution (DMSO diluted in sterile saline), together with an intravenous injection of nafamostat (3 mg/kg) or vehicle (sterile saline). Fresh brain was collected 6-h post-challenge. Relative expression of TNF (**A**), IFN-γ (**B**), CXCL1 (**C**) and CXCL10 (**D**) in the prefrontal cortex were determined by qPCR. Naïve animals were included to establish baseline (dotted line). Data presented as mean ± SEM, *n* = 4–10/group, and analysed by two-way ANOVA, ***p < 0.001, ****p < 0.0001 main effect, ^#^*p* < 0.05, ^##^*p* < 0.01, ^####^*p* < 0.0001 control vs. R848 with Sidak’s post hoc test

The expression of pro-inflammatory genes in the frontal lobe of the brain was examined to understand the impact of nafamostat on R848-induced central cytokine production. The frontal lobe was used in this study as previous reports have demonstrated inflammatory changes in this brain region in association with sickness behaviour [12, 32]. Moreover, we have shown that ameliorating inflammation in the prefrontal cortex rescues animals from behavioural deficits [33], suggesting this brain structure may drive sickness behaviour. Expression of TNF (Fig. 7A; two-way ANOVA, interaction *p* = 0.952, challenge *p* < 0.001, treatment *p* = 0.788), IFN-γ (Fig. 7B; two-way ANOVA, interaction *p* = 0.794, challenge *p* < 0.0001, treatment *p* = 0.731), CXCL1 (Fig. 7C; two-way ANOVA, interaction *p* = 0.540, challenge *p* < 0.001, treatment *p* = 0.546) and CXCL10 (Fig. 7D; two-way ANOVA, interaction *p* > 0.999, challenge *p* < 0.0001, treatment *p* = 0.999) were all significantly increased with the R848 challenge. Nafamostat had no effect on the expression of any of the genes examined here.

## Discussion

Here, we have shown that R848 administration induces marked changes in the expression of central and peripheral cytokines, which is associated with the induction of sickness behaviour, an acute febrile response, and an acute leukocytopenia. The administration of nafamostat had no significant effect on food intake, the SPT or exploratory behaviour when challenged, and failed to alter central cytokine production. However, nafamostat did reduce hepatic cytokine production and normalised the levels of circulating leukocytes.

Currently, the polyI:C model is widely used as a TLR3 activation model to mimic dsRNA virus infection. In particular, it has gained the most traction in modelling gestational immune activation. However, the model can generate variable results that are dependent on the source and the molecular weight of the polyI:C used, which, coupled with age-related changes, seems to have a profound effect on the immunological outcome [39, 40]. SARS-CoV-2 can also activate TLR3 during viral replication, but mechanisms appear to have evolved to enable the virus to suppress TLR3-TRIF signalling [41]. In comparison to SARS-CoV and MERS-CoV, whole-genome sequencing of SARS-CoV-2 has revealed that TLR7 is likely to be of particular importance in pathogenesis of SARS-CoV-2 as it has more ssRNA motifs that could bind to TLR7 [42]. Furthermore, loss-of-function variants of X-chromosomal TLR7 have been identified in young male patients with severe COVID-19 [43]. Thus, the use of R848 presents a useful opportunity to understand the impact of TLR7 activation on behaviour and the systemic inflammatory response in viral illnesses.

It has previously been reported that R848, a TLR7/8 agonist, can induce an acute sickness behaviour phenotype [22, 25]. Consistent with these previous studies, we also observed that R848 induced an increase in body temperature, indicative of fever, and a decrease in food intake. However, sickness behaviour encompasses a wider range of behavioural and physiological changes and thus we further characterised the behavioural response to the challenge. Sucrose preference has been widely adopted to evaluate the ability of a rodent to experience pleasure [29, 44, 45], as rodents have been demonstrated to drink sweetened water avidly [46]. In this study, total sucrose intake was decreased by the challenge, however there was no significant effect on sucrose preference, suggesting that R848, at this early time point, does not induce anhedonia. Although, most models of infection-associated sickness behaviour exhibit anhedonia after a 12–24 h time period [47], and so it is plausible that an R848-induced decrease in sucrose preference may become evident at later time points.

In contrast to the SPT, exploratory behaviour was significantly affected by R848; the number of grid crossings/minute was decreased in R848-challenged animals, which is consistent with other inflammatory models [48–50], such as those employing the classical LPS-CD14-TLR4 challenge [51]. Whilst a total decrease in motility could be a confounder, and change the interpretation of this result, no differences in the FST were observed, indicating there are no locomotor deficits as a result of the challenge. Therefore, we conclude that the decrease in grid crossings is due to R848-induced sickness behaviour, specifically. Consistent with this, a decrease in rearing during the same OF test was observed, which has been shown to be independent of motility [36, 47]. It must be acknowledged that the OF test is also a valid measure of depressive-like behaviour, which is distinct from sickness behaviour, although there is significant overlap [52]. However, given the early time point at which the OF was performed (4-h post-challenge), this response should be attributed to sickness behaviour, rather than a depression-like phenotype, in line with previous studies [48, 53, 54]. Analysis of position in the open field revealed that the R848-treated animals chose to spend less time in the centre of the field, which suggests that R848 had an anxiogenic effect. Increased anxiety has been reported in LPS models [51], which can be exacerbated by stress [12, 29], and is associated with central TNF expression, as was detected here. Collectively, for R848, the behavioural changes observed are indicative of a sickness behaviour phenotype, and this challenge provides a useful model of ssRNA virus-induced sickness behaviour.

Analysis of the FST data did not reveal any R848-mediated effect, as both latency to float and total float time were both unaffected by the challenge. This is, perhaps, not surprising as the FST is a measure of helplessness [55], which is not often associated with sickness behaviour. Indeed, previous studies with an LPS challenge were similarly unable to demonstrate consistent changes in this test [56]. The FST is widely used to measure the efficacy of potential anti-depressant therapies [55], however interpretation of the results is controversial. Whilst increased immobility is thought to reflect a depressive-state, this response could similarly suggest habituation and learning [57], which are often perceived as an adaptive behavioural response. As such, the FST may not be a good indicator of sickness behaviour, however, there was a significant main effect of nafamostat on the total float time, suggesting that nafamostat does have some unexpected behavioural effects. Instead, this FST result has served as a helpful control for our other behavioural outcomes. Given that there was no effect of the R848 challenge, we have shown there are no locomotor deficits in this model, which could confound our OF results (as discussed above). Similarly, with no differences in the FST, we can attribute significant differences in the OF, SPT and food intake tests to sickness behaviour, rather than a depression-like phenotype.

Leukocytosis is commonly observed in response to infection. Therefore, it was surprising to note that the R848 challenge induced depletion of circulating leukocytes, and, more specifically, in circulating lymphocytes. Although unexpected, this is not a unique observation; a decrease in blood lymphocytes has been reported in patients and in animal models of acute viral infection [58–60], including after TLR7 stimulation [61, 62]. Interestingly, clinical studies have reported a decrease in CD4+ and CD8+ T lymphocyte counts in COVID-19 patients [63, 64], and, therefore, the effects of R848 could be recapitulating the lymphopenia observed in this disease. The mechanism by which this depletion occurs is unclear, although it may be owing to widespread sequestration in peripheral organs. It is known that leukocytes infiltrate peripheral organs to locate the source of infection [51, 60], as such our result may reflect this infiltration, which may be occurring more rapidly than cell replacement from lymphoid organs. Alternatively, lymphocytes may be undergoing increased cell death. Diao et al. [64] showed that remaining T-cells from COVID-19 patients had increased levels of PD-1, a protein surface marker that promotes apoptosis. To explore this, immunostaining for neutrophils, macrophages and lymphocytes was performed on peripheral and central tissues. There was significant infiltration of neutrophils into the livers and brains of R848-treated mice. By contrast, macrophage density in the brain and lung was unaffected, but decreased in the liver. Lung B lymphocyte density was similarly decreased, which has been reported previously after topical treatment with TLR7 agonist imiquimod [65]. This is consistent with previous reports showing that leukocyte emigration into tissues is not dramatically affected by R848 challenge [61], which suggests infiltration of organs is not responsible for the R848-induced decrease in blood leukocytes. However, tissue sequestering cannot be completely ruled out, simply that the cells did not infiltrate the brain, liver or lung. Interrogation of other organs, such as the spleen, would need to be completed. Critically, the depletion of circulating leukocytes was normalised by treatment with nafamostat. Given that the magnitude of lymphocyte depletion was associated with disease severity [64], this result suggests that nafamostat treatment may reduce the severity of COVID-19 and improve patient outcomes.

The magnitude of the systemic inflammatory response was evaluated by measuring the relative expression of pro-inflammatory genes in the liver, lung and brain [53]. In all organs, the R848 challenge induced a significant increase in TNF, IFN-γ, CXCL1 and CXCL10. In addition, hepatic expression of the APPs SAA-2 and CRP were elevated. These inflammatory mediators have long been associated with viral infection and the induction of sickness behaviour [66]. Indeed, increased expression of these cytokines have been reported in animal models and humans with ssRNA infection; for example, mice infected with SARS-CoV-2 exhibited increased IFN-γ and CXCL10 expression in the lung at levels comparable to COVID-19 patients [67, 68]. TLR7 is strongly expressed by macrophages [69]; it seems probable that it is the resident tissue macrophages that are responsible for producing these cytokines [70]. Han et al. [71] demonstrated that infection of ssRNA40 induced expression of TNF in macrophages, which corroborates our hypothesis. This would also account for the differential expression levels between the organs, as there is a greater density of macrophages in the liver than the lung. Certainly, challenge with R848 appears to closely mimic the cellular and molecular response to ssRNA infection.

It is of note that R848 has been shown to have anti-viral properties, but, given the data reported here, it can also be used to model the impact of TRL7 activation during ssRNA virus infections. TLR7 agonists have been used therapeutically to treat herpes simplex virus and hepatitis C, due to their ability to provoke an IFN-mediated adaptive immune response to the virus [72]. Similarly, R848 was been shown to have anti-tumour abilities [73] and has been used as an adjuvant in vaccine development [74]. As in most instances of immune activation, the role of TLR7 signalling appears to be context dependent. In this study, we were keen to understand the nature of the acute inflammatory response induced by TLR7 activation in the absence of active virus infection. This is particularly pertinent now that it has been shown that ssRNA for the SARS-CoV-2 virus is detectable long after infectivity is lost [75, 76], and thus the impact of ‘viral litter’ on the persistent inflammatory response in the context of long COVID, and other post viral syndromes, is now of considerable interest. However, it is of note that extended exposure of TLR receptors to their ligand can result in tachyphylaxis. LPS, as an indirect TLR4 agonist, is known to induce acute sickness behaviour, but can also induce tolerance and be protective [77]. This has also been reported following extended R848 treatment [22]; the impact of the chronic administration of R848 on behavioural outcomes would be an interesting topic for a downstream study.

Here, we were also able to show that nafamostat had a peripheral anti-inflammatory effect; hepatic TNF and IFN-γ expression, induced by R848, was significantly ameliorated by nafamostat treatment, as well as CXCL1 to a lesser extent. As this model does not employ a live virus, and, instead, triggers a downstream immune response via TLR7/8 activation, this highlights the utility of nafamostat as a broad-spectrum serine protease inhibitor that is able to suppress inflammation, independent of viral cell entry. Certainly, serine proteases have been implicated as moderators of the inflammatory response by regulating cytokine and chemokine expression [78]. For example, neutrophil-derived Cathepsin G is a serine protease, which stimulates IFN-γ production and cleaves membrane-bound TNF-α [79], although it is acknowledged that the IC50 is much higher for Cathepsin G than for TMPRSS2. Many enzymes of the complement cascade, including C1r and C1s, are also in the serine protease family [80]. Therefore, nafamostat may be acting upon any number of alternative endogenous enzymes to cause the observed anti-inflammatory effect.

Despite the attenuation of the hepatic inflammatory response, nafamostat treatment had no effect on brain cytokine expression. We, and others, have shown that a peripheral inflammatory challenge induces a central cytokine response in the CNS [47]. In keeping with this, systemic administration of R848 induced pro-inflammatory gene expression in the prefrontal cortex of the brain, including a 400-fold increase in CXCL10 expression. Although, it remains unknown precisely how signals from the periphery result in central cytokine production [34] or whether, in this case, R848 is able to activate central TLR7 directly. As it has been shown that nafamostat is unable to cross the BBB [81], it seems likely that it is the absence of any CNS bioavailability that resulted in no change in central cytokine expression observed in this study. Nafamostat is also metabolised quickly and cleared in vivo with a half-life of 23 min [82], which would further prevent appreciable accumulation of nafamostat in the brain. Despite this, we have previously shown that inhibition of cytokine signalling in the periphery can attenuate sickness behaviours induced by inflammation in the brain [54, 83], and so we might have expected some attenuation of the response. Although, timing may be an issue. Duan et al. [6] showed that nafamostat reduced expression of pro-inflammatory cytokines in the spinal cord after traumatic injury, however authors evaluated levels 24-h post-injury, whilst in this study, brains were collected 6-h post-challenge. Additional later time points, or a multi-dose treatment regime could be explored to investigate this.

Clinical trials investigating the efficacy of nafamostat in the treatment of COVID-19 are currently underway. Whilst its primary mechanism of action is proposed to be inhibition of TMPRSS2 and subsequent viral entry, data in this study suggest that nafamostat has additional immunological effects, which may be beneficial in the treatment of COVID-19. Nafamostat may also limit disease-associated coagulopathy and improve patient survival. COVID-19 patients exhibit clotting disorders, which have been associated with the severity of the disease and are predictive of death [84, 85], and nafamostat inhibits the coagulation cascade [86]. Indeed, case reports have identified improvements in COVID-19 patients with DIC when treated with nafamostat [4, 87]. Finally, as highlighted previously, nafamostat is currently approved to treat pancreatitis, and so it has a proven safety record. This, combined with its known pharmacokinetics, indicates that repurposing it for the treatment of COVID-19 could be rapid and straightforward.

## Conclusion

A single injection of the TLR7/8 agonist R848 generates systemic and central inflammation and sickness behaviours, which is amenable to therapeutic intervention. Given that R848 is a small molecule agonist, and not a live virus, this model provides a safe and simple tool to investigate therapy designed to ameliorate inappropriate and excessive TLR7 activation, and to understand the impact of TLR7 activation on affect. The serine protease inhibitor nafamostat was able to attenuate peripheral inflammation and normalise circulating leukocyte numbers in this model. As these results were achieved in the absence of viral entry, we propose that nafamostat may have useful anti-inflammatory effects, which may be advantageous in the treatment of viral infections, including COVID-19.

## Data Availability

All data generated during and/or analysed during the current study are included in this published article.

## Abbreviations

ANOVA: Analysis of variance
APR: Acute-phase response
COVID-19: Coronavirus disease 2019
DIC: Disseminated intravascular coagulation
dsRNA: Double-stranded RNA
MERS: Middle East respiratory syndrome
PRR: Pattern recognition receptor
SARS: Severe acute respiratory syndrome
SARS-CoV2: Severe acute respiratory syndrome coronavirus 2
SEM: Standard error of the mean
ssRNA: Single-stranded RNA
TLR: Toll-like receptor
TMPRSS2: Type 2 transmembrane serine protease
